# Biological and structural characterization of the Type 3 fimbrial subunit MrkA from *Klebsiella pneumoniae*

**DOI:** 10.1002/pro.70343

**Published:** 2025-10-21

**Authors:** Valentina Monaci, Davide Oldrini, Gianmarco Gasperini, Lucia Banci, Francesca Cantini, Francesca Micoli

**Affiliations:** ^1^ Magnetic Resonance Center – CERM University of Florence Florence Italy; ^2^ Department of Chemistry University of Florence Florence Italy; ^3^ GSK Vaccines Institute for Global Health (GVGH) Siena Italy; ^4^ GSK Siena Italy

**Keywords:** *Klebsiella pneumoniae*, NMR structure determination, protein antigen, self‐complemented MrkA monomer, Type 3 fimbriae, vaccine design

## Abstract

*Klebsiella pneumoniae* is a Gram‐negative opportunistic pathogen responsible for a wide range of community‐associated and hospital‐acquired infections and a major cause of neonatal sepsis in low‐ and middle‐income countries. The pathogen's surface fimbriae, particularly the Type 3 fimbriae, are critical for bacterial adhesion, biofilm formation, and host defense evasion. MrkA, the pathogen's major Type 3 fimbrial subunit, has a structural function in fimbrial assembly, but its three‐dimensional structure remains to be fully elucidated. In this study, we utilized solution‐state nuclear magnetic resonance spectroscopy to elucidate the structure of MrkA, leveraging previously reported chemical shift assignments of a designed self‐complementing monomeric protein. Additionally, we confirmed the ability of monoclonal antibodies, capable of recognizing MrkA oligomers on wild‐type *Klebsiella* bacteria, to bind the recombinant MrkA protein. Our findings contribute to the evaluation of MrkA as a potential target in vaccine development against *Klebsiella pneumoniae* infections.

## 1 INTRODUCTION

Kp is a Gram‐negative opportunistic pathogen that has been linked to a broad array of community‐associated and hospital‐acquired infections, including pneumonia, catheter‐associated urinary tract infections, bacteremia, and surgical wound infections (Wen et al., 2021; Zaidi et al., 2009). Additionally, Kp has recently been identified as a leading cause of neonatal sepsis in low‐ and middle‐income countries, which has led to growing public health concern (Kumar et al., 2023; Ma et al., 2024; Verani et al., 2024). The increasing antimicrobial resistance among Kp isolates and the emergence of hypervirulent Kp strains have further increased the threat posed by this pathogen (Arato et al., 2021; Dangor et al., 2024; Rice, 2008; Sati et al., 2025). Currently, no licensed Kp vaccines are available. Several vaccine targets have been described over the past few decades. Kp isolates commonly display on their surface two different classes of lipopolysaccharides (O‐antigens, O‐Ag) and capsular polysaccharides (K‐antigens, K‐Ag) and fimbrial adhesins. Both K‐Ag and O‐Ag contribute to pathogenicity and form the basis for the identification of isolates, or serotyping. A K‐Ag based vaccine strategy could be complex because of the high variability and structural diversity of such polysaccharides (Assoni et al., 2025). Therefore, less diverse polysaccharides (e.g., O‐Ag) or highly conserved surface proteins (e.g., Type 3 fimbrial subunit MrkA) are recently gaining attention as alternative vaccine targets (Nonne et al., 2025; Whitfield et al., 2025).

Type 3 fimbriae are among the most widespread in Kp. These helix‐shaped, membrane‐bound, adhesive structures play a crucial role in bacterial adhesion to host cells and abiotic surfaces, as well as in biofilm formation (Langstraat et al., 2001; Tarkkanen et al., 1997). Biofilm formation is critical in many bacterial infections and contributes to the ability of bacteria to overcome host defense mechanisms. The Type 3 fimbrial appendage is primarily composed of the major fimbrial subunit MrkA, a 20‐kDa protein that polymerizes to form a pearl‐necklace structure, and MrkD, which is located at the tip of the fimbriae. MrkA has been identified as a common protein antigen expressed by the majority of Kp strains (Wang et al., 2016). It shows a high degree of sequence conservation among representative members of the Enterobacteriaceae family and, as a surface protein of Kp, is accessible to antibodies. The immunogenicity of Type 3 fimbriae was explored in a challenge study using a murine model of acute pneumonia (Lavender et al., 2005). A strong immune response was detected in all animals receiving purified Type 3 fimbriae as an immunogen. Interestingly, immunization with purified Type 3 fimbriae resulted in complete protection against a lethal dose of bacteria (Lavender et al., 2005). Furthermore, using a target‐agnostic approach, Wang et al. identified protective antibodies against Kp: monoclonal antibodies (mAbs, such as Kp3, St1C1, and St4C6) were isolated from both phage display and hybridoma platforms by functional screening for in vitro opsonophagocytic killing activity. Differently from St1C1 and St4C6, which recognize only the monomeric form of MrkA with different epitopes as elucidated by peptide mapping, Kp3 binds a conformational epitope that exists predominantly in the oligomeric form of MrkA (Wang et al., 2017). Importantly, all three anti‐MrkA antibodies elicited in vivo protections against Kp strains (Wang et al., 2016, 2017). Type 3 fimbriae are encoded by the *mrk* gene cluster (*mrkABCDF*). This cluster is comprised of five genes encoding the structural and assembly components of the appendages, and highly conserved within Kp and *Enterobacter* species (Gerlach et al., 1988, 1989; Old et al., 1985). The assembly of the Type 3 fimbriae is chaperone/usher pathway mediated, as well documented for *Escherichia coli* (Walczak et al., 2014). All structural subunits of MrkA are homologous proteins sharing an immunoglobulin (Ig)‐like fold. After secretion of MrkA subunits into the periplasm, they are recognized by the fimbrial assembly chaperone MrkC (usher) that stays bound to the native subunits and delivers them to the outer membrane assembly platform MrkD, where the subunits are incorporated into the growing fimbriae. Neighboring subunits in the fimbriae interact via a mechanism termed donor strand complementation in which the incomplete Ig‐like fold of each subunit, lacking the seventh (last) β‐strand of the Ig fold, is completed by an N‐terminal extension (donor strand) of the following subunit that inserts in an antiparallel orientation relative to the sixth strand. In MrkC‐subunit complexes, the lacking strand is provided to the subunit by an extended segment (donor strand) of MrkC that, however, inserts in a parallel orientation relative to the subunit's C‐terminal strand. During subunit incorporation into the growing fimbriae, the donor strand of the chaperone is displaced by an N‐terminal donor strand extension of the next, incoming subunit (Walczak et al., 2014). MrkA, rather than the adhesion molecule MrkD, has been identified as a key contributor to biofilm formation (Langstraat et al., 2001). The structural characterization of MrkD has been determined by x‐ray crystallography (PDB ID: 3U4K) (Jagnow & Clegg, 2003); however, detailed structural information on MrkA remains limited. To address this knowledge gap, we performed an in‐depth structural characterization of MrkA using solution‐state nuclear magnetic resonance (NMR) spectroscopy, taking advantage of the published complete chemical shifts assignment of self‐complemented MrkA monomer (Monaci et al., 2024). Self‐complemented MrkA monomer is, hence, considered a model for studying the structure and stability of MrkA in the context of the Type 3 fimbriae. Structural studies will contribute to the use of MrkA as a candidate antigen for Kp vaccines.

## 2 RESULTS

### 2.1 MrkA conservation

A BLAST analysis identified MrkA as a highly conserved protein across a range of 38,939 *Klebsiella* strains annotated in the Pasteur Institute *Klebsiella* database, establishing its conservation at the species level and confirming what was previously published at the *Enterobacteriales* order level. MrkA was identified considering >85% identity and >90% coverage as thresholds at the protein sequence level and found in 36,877 isolates (94.7% of the database). Average protein conservation resulted to be 95.7% in all *Klebsiella* isolates (including 10 *Klebsiella africana* isolates, 35,311 Kp isolates, 3 Kp subsp. *rhinoscleromatis* isolates, 266 *Klebsiella quasipneumoniae* subsp. *quasipneumoniae* isolates, 520 *Klebsiella quasipneumoniae* subsp. *similipneumoniae* isolates, 15 *Klebsiella quasivariicola* isolates, 7 *Klebsiella variicola* isolates, 21 *Klebsiella variicola* subsp. *tropica* isolates, and 724 *Klebsiella variicola* subsp. *variicola* isolates) (see Table S1).

### 2.2 Self‐complemented MrkA monomer design and production

**FIGURE 1:**
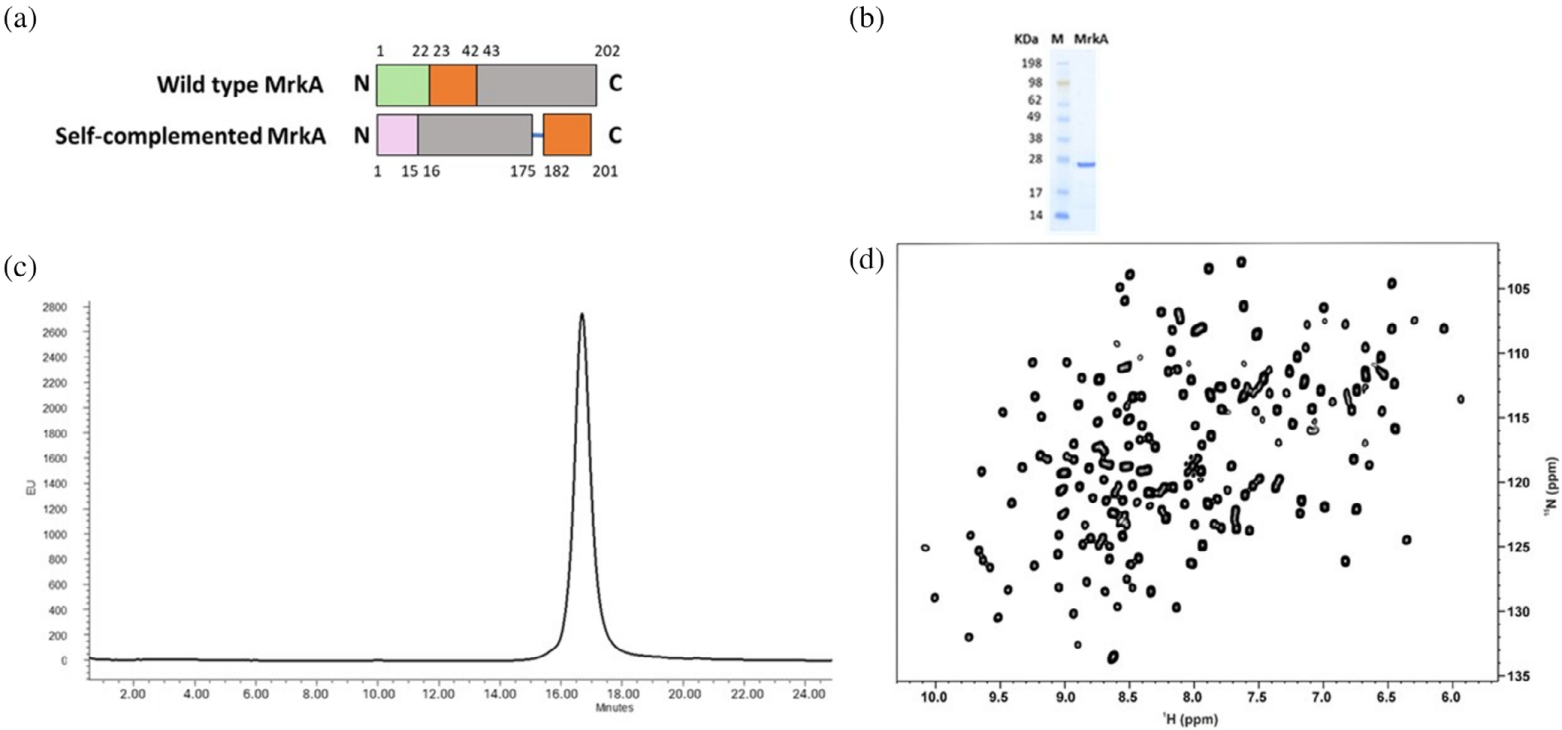
(a) Wild‐type and self‐complemented MrkA fusion construct. Schematic representation of the self‐complemented MrkA monomer, in which the donor strand (in orange) is at the C‐terminus, preceded by a glycine stretch (in blue) and at the N‐terminus there is the 10xHis tag (in pink). The first 22 residues of the wild type form represent the leader sequence of the protein (in green). Ser 42, Gln 202, and the N‐ terminus stretch (Ala 23–Ser 42) of the wild‐type protein matches Ser 15, Gln 175, and the C‐terminus stretch (Ala 182–Ser 201) in the self‐complemented MrkA construct. (b–d) Biochemical and biophysical results of the self‐complemented MrkA monomer. SDS‐PAGE (b) and HPLC‐SEC chromatogram (c) of purified MrkA confirmed its monomeric form. (d) Folding analysis of MrkA by nuclear magnetic resonance. Bidimensional HSQC spectrum of MrkA in 50 mM sodium phosphate, 100 mM NaCl pH 7.0 (950 MHz, 298 K). HPLC‐SEC, High‐Performance Liquid Chromatography—Size Exclusion Chromatography; HSQC, ^1^H‐^15^N Heteronuclear Single Quantum Coherence.

To investigate the structure of MrkA, we previously designed a self‐complemented MrkA monomer (Monaci et al., 2024), further referred to as MrkA. The monomer was produced as recombinant soluble protein in *E. coli* with a yield of 100 mg/L of culture (Figure 1a and Table S2). Despite the formation of a few aggregates, MrkA was isolated using an additional step of Size Exclusion Chromatography (SEC) purification with a Superdex75 column after a HisTrap FF affinity step. MrkA was obtained with high purity as verified by sodium dodecyl sulfate‐polyacrylamide gel electrophoresis (SDS‐PAGE gel) and High‐Performance Liquid Chromatography—Size Exclusion Chromatography (HPLC‐SEC) analysis (Figure 1b,c). Moreover, the ^1^H‐^15^N Heteronuclear Single Quantum Coherence (HSQC) spectrum by solution NMR showed well‐dispersed resonances indicative of an overall well‐folded protein (Figure 1d).

### 2.3 Evaluation of MrkA monomer antigenicity

**FIGURE 2:**
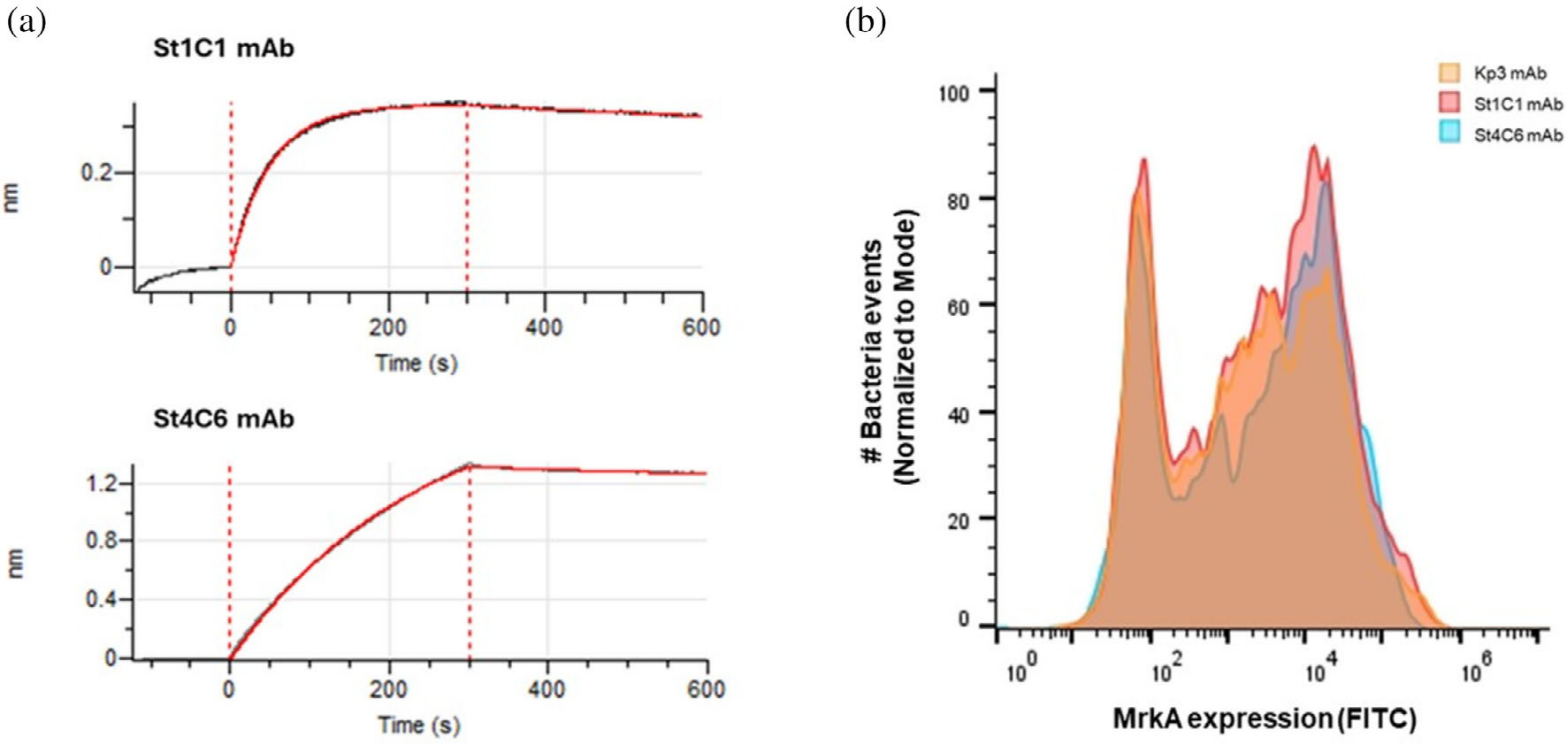
(a) Biolayer interferometry results. Binding response curves of MrkA with St1C1and St4C6 monoclonal antibodies (mAbs), using anti‐human IgG Fc Capture (AHC) or nickel‐charged tris‐nitrilotriacetic (Ni‐NTA) biosensors, respectively, with MrkA concentration of 5 (St1C1) or 100 (St4C6) μg/mL and mAb concentration of 125 or (St1C1) or 66.7 (St4C6) nM. (b) Flow cytometry results on *Kp* strain NCTC9135 with different mAbs. mAbs, monoclonal antibodies.

To verify that the MrkA design yielded a native‐like protein, available human functional St1C1 and St4C6 mAbs (referred to, respectively, as Clone 1 and Clone 6 in Wang et al., 2017), were used to confirm the antigenicity of the purified construct by biolayer interferometry (BLI). BLI results showed that St1C1 and St4C6 mAbs have high affinity for the self‐complemented MrkA monomer as for the wild‐type MrkA form (Wang et al., 2017), with affinity constant (*K*_D_) values of 10^−9^ M (Figure 2a and Table 1). Overall, the kinetic constants indicated that the two mAbs bind the protein with similar association and dissociation rates, forming highly stable complexes as evident from the plotted curves in Figure 2a. Importantly, both mAbs were verified to bind native Type 3 fimbriae produced by Kp bacteria via flow cytometry (Figure 2b). For both experiments, Kp3 mAb was included as an additional control and showed exclusive binding on native MrkA oligomers (as verified by fluorescence‐activated cell sorting [FACS]) and not on recombinant MrkA monomer (as verified by BLI, where it was used as a negative control), as previously reported. No significant differences among the three antibodies were observed in FACS analysis.

**TABLE 1:** Kinetic constants of anti‐MrkA monoclonal antibodies by biolayer interferometry.

mAb	*K*_on_ (1/Ms)	*K*_on_ error	*K*_off_ (1/s)	*K*_off_ error	*K*_D_ (M)	*K*_D_ error
St1C1	1.585E05	2.489E02	2.257E‐04	2.099E‐06	1.424E‐09	1.343E‐11
St4C6	6.178E04	1.886E02	1.281E‐04	2.193E‐06	2.074E‐09	3.605E‐11

*Note*: Kinetic constants (*K*_on_, *K*_off_, *K*_D_) of anti MrkA human functional monoclonal antibodies St1C1 and St4C6 tested against MrkA in biolayer interferometry experiments.

### 2.4 MrkA structure determination by solution NMR spectroscopy

**FIGURE 3:**
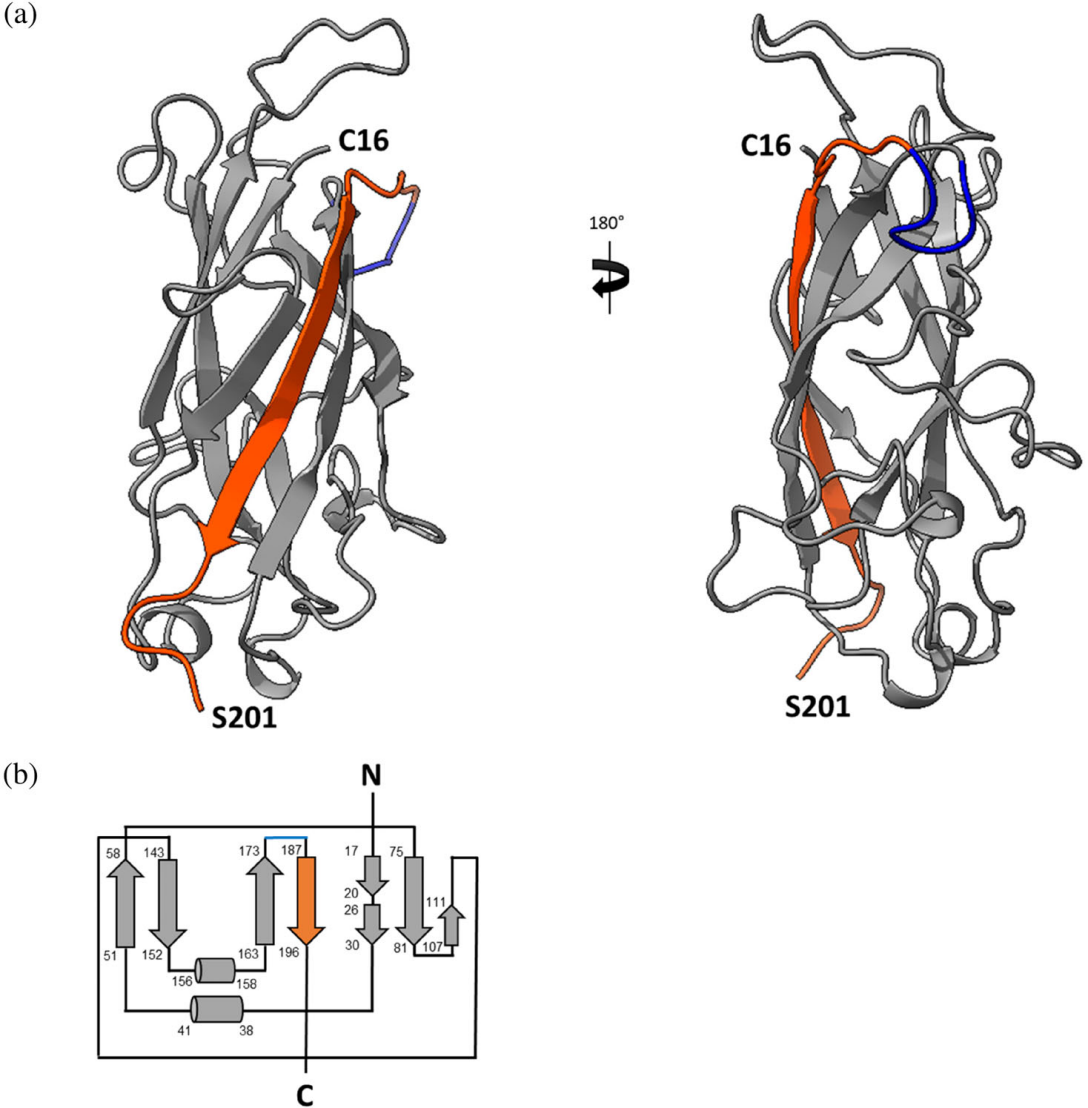
(a) Three‐dimensional molecular structure of MrkA family 20 conformers. In orange the donor strand and in blue the glycine stretch. (b) MrkA topology of NMR structure. Representation of secondary structure MrkA topology by arrows (β‐sheet) and cylinders (α‐helix) with their corresponding sequence number on the left. NMR, nuclear magnetic resonance.

**FIGURE 4:**
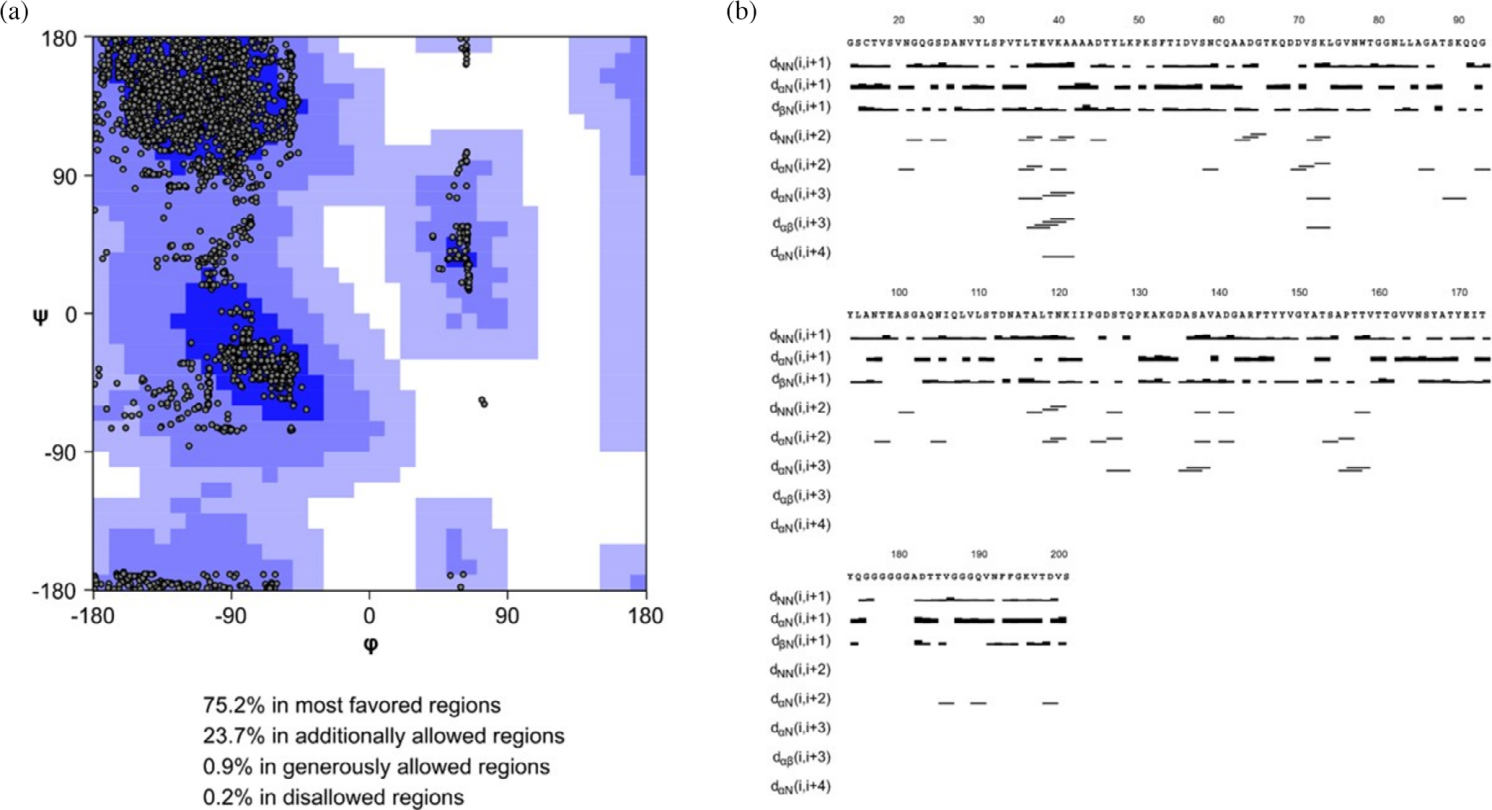
(a) Ramachandran plot with usual distribution of dihedral angles (*ϕ*, *ψ*). Values related to TALOS‐N prediction are figured by dots. (b) MrkA NOEs pattern. Sequential and medium‐range NOEs restrains for secondary structure elements of MrkA.

**FIGURE 5:**
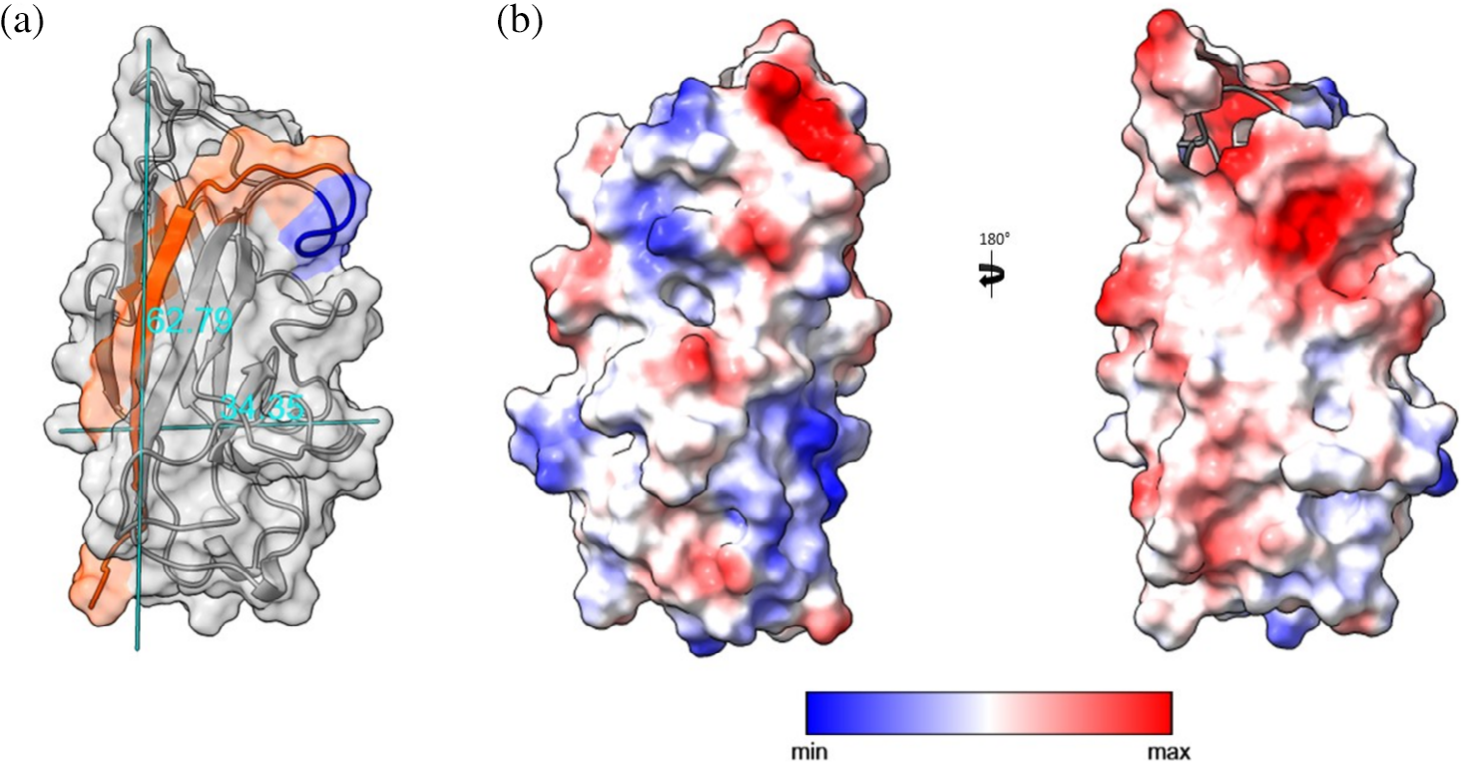
(a) Identification of height and diameter (Å) of MrkA structure calculated by ChimeraX. (b) MrkA electrostatic surface potential. The electrostatic surface potential is mapped on a solid surface representation of MrkA (Chain A) using ChimeraX (transparency 70%). According to the legend, in (b) representation, red region are negative and blue are positive.

**FIGURE 6:**
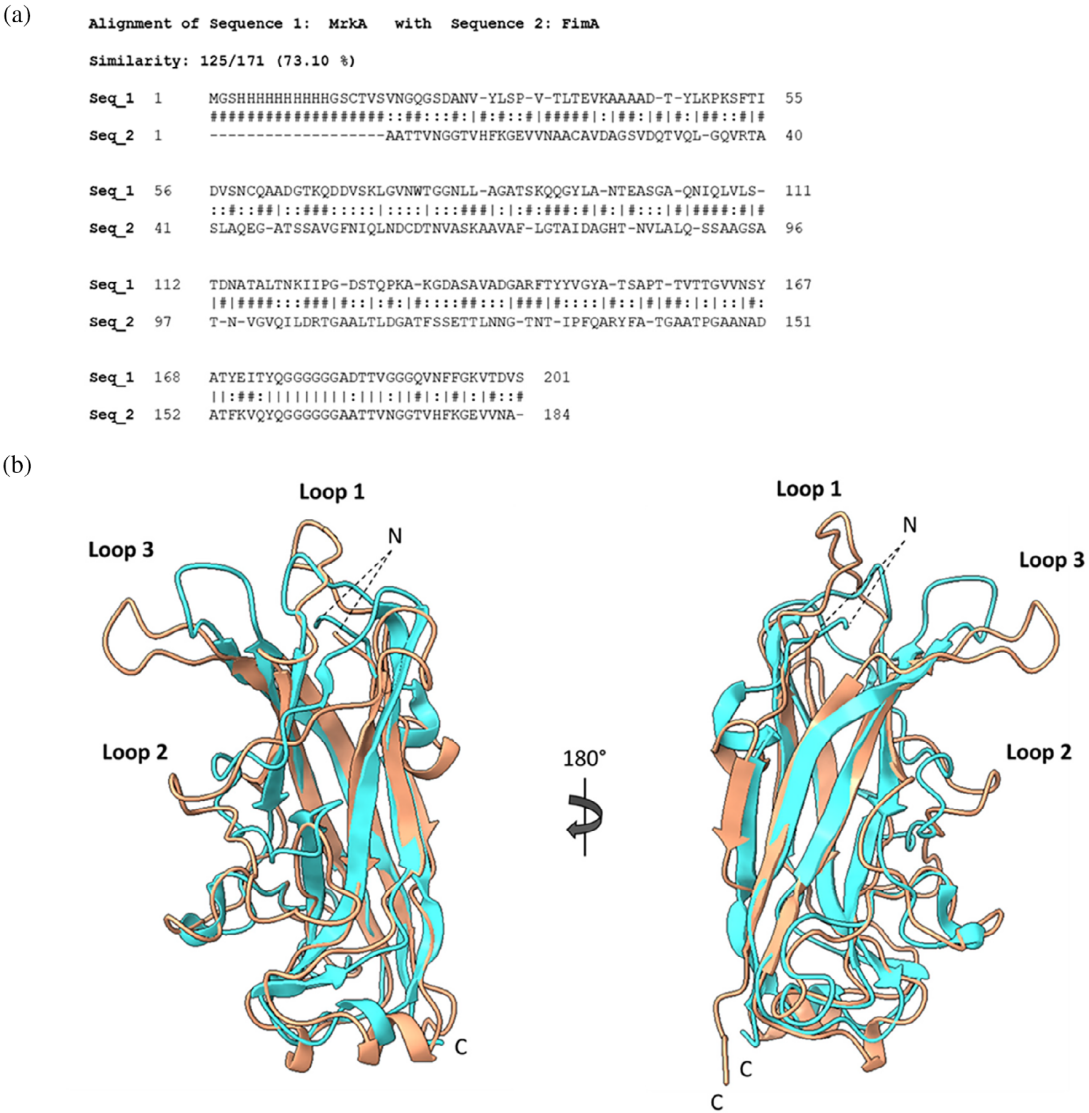
(a) BLAST analysis of MrkA and FimA. Sequence allignment of MrkA (Sequence 1) and FimA (Sequence 2) obtained through BLAST analysis resulted in 73.1% of identity. Residues that are strictly conserved have a line (|), residues well conserved within a group are semicolon (:) and other residues are hash‐mark (#). Gaps in sequences are represented by the minus symbol (−). (b) Analysis of MrkA and FimA's structures. Structure comparison of MrkA (salmon) with FimA of *Escherichia coli* (PDB ID: 2JTY, cyan) resolved with solution NMR, highlighting major differences in Loops 1, 2 and 3 at RMSD 1.2 Å. NMR, nuclear magnetic resonance; RMSD, root mean square deviation.

A total of 2248 meaningful proton‐proton distance restraints (Figure S1 and Table S3) out of 2416 assigned cross peaks, together with 134 *ϕ* and 135 *ψ* backbone dihedral angle restraints, derived from TALOS‐N program (Shen & Bax, 2013), were used for the structure calculations. Distance constraints and dihedral angles have been submitted to the PDB database together with the family of the 20 lowest target function conformers after their refinement with AMBER (Case et al., 2005) in explicit water solvent (PDB ID: 9HW9). Statistical analysis of the AMBER‐refined family of conformers is reported in Table S3. The root mean square deviation (RMSD) of the conformers of the family to the mean structure (for stretches 16–175 and 181–201) was 1.08 ± 0.21 and 1.46 ± 0.21 for the backbone and heavy atoms, respectively. The most disordered regions are those involving residues 60–70 and 176–180 due to the small number of Nuclear Overhauser Enhancements (NOEs) and, for the latter segment, also to the lack of backbone and/or side chain assignments (Figure S2). The solution NMR structure of MrkA and its corresponding topology are shown in Figure 3. In the Ramachandran plot (Figure 4a), 75.2% of dihedral angles were distributed in the most favored regions and the remaining 23.7% in additionally allowed regions. The distribution of sequential and medium‐range NOEs along the sequence (Figure 4b) aligns with the secondary structure elements predicted by TALOS‐N. The MrkA solution structure consists of 8 parallel and antiparallel β‐strands organized in an Ig‐like β‐sandwich fold. The MrkA structure has a height of 62.79 Å and a main chain diameter of ∼34 Å (Figure 5a), which is stabilized by a strong hydrogen bond network linking all the β‐strands. The core was characterized by several hydrophobic interactions, which involve buried aromatic and aliphatic residues (Cys 16, Val 29, Val 34, Val 39, Leu 48, Phe 53, Ile 55, Val 57, Leu 74, Trp 78, Gly 80, Leu 95, Tyr 94, Ala 106, Leu 108, Val 109, Leu 110, Ile 122, Pro 124, Ala 132, Phe 145, Tyr 147, Val 149, Tyr 151, Ala 152, Val 164, Ala 168, Ile 172, Tyr 174, Val 186, Phe 193). The obtained MrkA structure was compared with the solution‐state NMR structure of self‐complemented fimbrial subunit of Type 1 fimbriae FimA of *E. coli* (PDB ID: 2JTY) (Puorger et al., 2011). The two proteins share a 73.1% sequence identity, as calculated by BLAST‐P software (Altschul et al., 1997, 2005) (Figure 6a), which is reflected in structure similarity. However, by superimposing the two structures (the first family conformer for both, Chain A) with an RMSD of 1.2 Å, meaningful differences were observed for regions in correspondence of disordered fragments indicated as Loops 1–3, as shown in Figure 6b. To the best of our knowledge, no biological information about these regions is reported in the literature.

## 3 DISCUSSION

MrkA is an interesting surface protein antigen for *Kp* (Wang et al., 2016). Our BLAST analysis showed that MrkA is a highly conserved protein in the *Klebsiella* species, confirming previous reports (Wang et al., 2017). The Pasteur Institute *Klebsiella* database provided curated sequence information for a large number (*n* = 38,939) of *Klebsiella* isolates, making it an ideal resource for identifying conserved proteins, as in the case of MrkA. MrkA conservation and accessibility on the bacterial surface, as also confirmed in this study by FACS analysis with functional specific mAbs, make it a good potential candidate for broad vaccine coverage.

MrkA was designed as a monomeric form using the donor strand complementation approach, resulting in an improved soluble expression and yield of purified protein as compared to what was expected for its oligomeric form. Our biochemical characterization confirmed the production of MrkA monomer as pure and stable protein. Additionally, the design of self‐complemented MrkA monomer did not alter its folding or, importantly, the recognition and binding by anti‐MrkA human functional mAbs as verified by monodimensional NMR spectrum and BLI experiments, respectively. The overall structure of MrkA comprises a typical fimbrial subunit fold (Żyła et al., 2019), which is formed by β1‐β2‐α1‐β3‐β4‐α2‐β5‐β6‐α3‐β7‐β8. The solved NMR structure of MrkA showed that it is capable of intra‐molecular self‐complementation (β7–β8), in which the N‐terminal donor strand (β8) is inserted in the opposite orientation relative to that observed for inter‐molecular donor strand complementation in MrkA polymers (i.e., parallel to the C‐terminal strand of MrkA).

In conclusion, our results support the use of MrkA as a potential target for Kp vaccines. Indeed, understanding the conformational aspects of MrkA can guide the design of a stabilized antigen, supporting the development of a rationally engineered vaccine candidate with improved efficacy. The elucidated MrkA structure provides insights into epitope accessibility, antigen stability, and potential modifications to enhance its immunogenicity. It is anticipated that vaccine development for Kp will contribute to efforts to reduce the high morbidity, mortality, and hospital costs associated with Kp infection and antimicrobial resistance, especially in low‐ and middle‐income countries.

## 4 MATERIALS AND METHODS

### 4.1 BLAST analysis for MrkA conservation in *Klebsiella pneumoniae* database

The MrkA protein sequence was analyzed using the BLAST algorithm against the online BIGSdb *Klebsiella* Database (https://bigsdb.pasteur.fr/klebsiella/; Diancourt et al., 2005), an open database developed by the Pasteur Institute, designed to support the analysis of genomic data in *Klebsiella* species. The analysis was performed using BLOSUM62 as a scoring matrix and four cycles of a maximum of 10,000 alignment for each. A number of 38,939 different *Klebsiella* strains were investigated for determining MrkA coverage. The alignment sequences were filtered by alignment length and percentage of identity, respectively greater than 182% and 90%.

### 4.2 Design of self‐complemented MrkA monomer

Based on the work performed on fimbrial subunits such as *E. coli* FimA monomer (Żyła et al., 2019), the same strategy for the design of self‐complemented MrkA monomer was applied. More in detail, the N‐terminal donor strand (first 20 aa in the mature protein after leader sequence cleavage) was moved to the C‐terminus, preceded by a glycine stretch, in the opposite orientation relative to that observed for inter‐molecular donor strand complementation in wild type fimbrial polymers. Thus, the numbering of the protein is such that Ser 42, Gln 202, and the N‐terminus stretch, Ala 23–Ser 42, of the wild‐type protein (mrkA—Fimbrial Subunit Type 3—*Klebsiella pneumoniae* | UniProtKB | UniProt, https://www.uniprot.org/uniprotkb/P12267/entry), matches with Ser 15, Gln 175, and the C‐terminus stretch, Ala 182–Ser 201, in our sequence construct.

### 4.3 Expression, purification, and characterization of self‐complemented MrkA

The self‐complemented MrkA coding sequence was ordered at Twist Bioscience as a synthetic gene cloned into the expression vector pET29b(+) plasmid under the control of isopropyl‐β‐D‐1‐thiogalactopyranoside (IPTG)‐inducible T7 promoter. A 10× histidine tag (His–Tag) was added to facilitate purification by affinity chromatography. The plasmid was used to transform *E. coli* BL21 (DE3) competent cells (Thermo Fisher Scientific). Cell growth was performed in HTMC medium (glycerol 15 g/L; yeast extract 30 g/L; addition of 20 g/L K_2_HPO_4_, 5 g/L KH_2_PO_4_, 5 g/L MgSO_4_, and 1 M KOH) at 30°C with 180 rpm shaking. When the culture reached an OD600 of 0.8–1, 1 mM IPTG was added to induce protein expression, and the cells were incubated at 20°C for 20 h. Cells were harvested by centrifugation at 4500 rpm for 30 min at 4°C and lysed using CelLytic reagent (Sigma‐Aldrich), following the manufacturer's instructions. After incubation, the lysate was centrifuged, and the supernatant containing the soluble protein was diluted with 50 mM sodium phosphate, 500 mM NaCl, 30 mM imidazole pH 7.4. A first step of purification through Immobilized‐metal affinity chromatography was used to isolate the protein of interest containing the His‐Tag. The filtered supernatant was loaded in a HisTrap FF affinity chromatography column (Cytiva); the column was washed with an imidazole gradient and proteins eluted with 500 mM imidazole, pH 7.4. Peak fractions were pooled, concentrated by Amicon (Millipore), and applied to the second step of purification, a SEC. A Superdex 75 Increase prepacked column (Cytiva) was chosen with an isocratic elution in phosphate‐buffered saline (PBS) pH 7.4 at 0.5 mL/min. Peak fractions were pooled together and sterile filtered.

Purified MrkA was characterized by SDS‐PAGE gel. Next, 10 μL of sample + NuPAGE LDS sample buffer 4× (Invitrogen) and NuPAGE Sample Reducing Agent 10× (Invitrogen) were boiled for 3 min at 100°C and then loaded in NuPAGE Novex Bis‐Tris 4–12% gel. The SDS‐PAGE gel was run in 2‐morpholinoethanesulphonic acid (MES) buffer at 150 V for approximately 45 min and then stained with ProBlue Safe Stain (Giotto Biotech). SeeBlue Plus2 pre‐stained Protein Standard (Invitrogen) was used as the protein ladder. Secondly, 100 μL of the sample at the concentration of 100 μg/mL was analyzed by HPLC‐SEC using a TSKgel_G3000 Guardcolumn PWH 17 μm (TOSOH BIOSCIENCE) eluting in PBS with a flow rate of 0.5 mL/min (run time 70 min). Finally, a standard solution‐NMR 1D experiment of the protein was acquired to evaluate its folding. Five hundred microliters of the sample concentrated at 400 μM + 10% D20 was inserted in a 5‐mm NMR tube and ^1^H‐NMR spectrum was acquired at 500 MHz.

The production of ^15^N‐ and ^13^C‐^15^N‐MrkA was obtained using the same protocols of expression and purification of unlabeled protein. The growth was performed in ^15^N‐ and ^13^C‐^15^N ISOGRO media (Monaci et al., 2024).

### 4.4 Antigenicity evaluation of self‐complemented MrkA monomer

To confirm the antigenicity of the designed MrkA monomer, the BLI technique was used to assess the binding of two functional human mAbs, St1C1 and St4C6 (Wang et al., 2017), to the self‐complemented MrkA monomer. Both MrkA and mAbs were immobilized, respectively, on nickel‐charged tris‐nitrilotriacetic (Ni‐NTA) and anti‐human IgG Fc capture (AHC) biosensors (Sartorius). All kinetic assays on Octet‐R8 (Sartorius) were performed at 25°C using 96‐tilted well plates (Sartorius), and samples were diluted in freshly prepared PBS with Kinetic buffer, which contains PBS + 0.1% bovine serum albumin (BSA), 0.02% Tween20, and Kathon (Sartorius). After preliminary tests with the two biosensors and at different concentrations of MrkA and mAbs, the following conditions were used for the calculation of the affinity constant (*K*_D_) of each mAb. For St4C6, the Ni‐NTA biosensor was washed in PBS with Kinetic buffer to reach the baseline (200 s) and then submerged into wells containing MrkA at 100 μg/mL for 300 s (association step). After the recording of the second baseline (120 seconds), the antibody at a fixed concentration of 66.7 nM was allowed to bind for 300 s, and the tip was then immersed into assay buffer for 300 s for dissociation. For St1C1, the anti‐human IgG Fc biosensor was loaded with the antibody at 125 nM concentration, and interaction with MrkA at 5 μg/mL was measured with the same assay parameters previously described. Before fitting the response curves and determining kinetic parameters of binding using Octet Analysis Studio software version 12.2 (Sartorius), the resulting sensorgram curves were subtracted from the blank binding curves obtained. Constant values were extracted directly from Octet Analysis Studio (v12.2, Sartorius). Affinity constants (K_D_) were obtained by globally fitting association (300 s) and dissociation (300 s) phases to a 1:1 Langmuir binding model in Octet Analysis Studio v12.2 (Sartorius).

Kp strain NCTC9135 (K15:O4) obtained from Public Health of England was characterized for the presence of MrkA on its surface by flow cytometry (FACS) using St1C1, St4C6, and Kp3 mAbs as primary antibodies (Wang et al., 2016, 2017). Kp was grown overnight at 37°C in Luria–Bertani medium and then diluted to 0.05 OD_600_ and grown for about 1 h to reach the exponential growth phase. A total of 1 OD of bacteria was pelleted and washed with PBS at 4000×*g* for 10 min. After washing, bacteria were fixed with 4% (w/v) formaldehyde for 30 min at 4°C. After that, bacteria were washed with PBS + 1% (w/v) BSA, plated in a round‐bottom 96‐well plate (100 μL/well, Thermo Fisher Scientific), and incubated with mAbs (100 μL/well) at a dilution of 1:300 in PBS–BSA for 1 h. Finally, samples were incubated with Alexa Fluor 488 goat anti‐human IgG diluted 1:500 in PBS–BSA for 30 min, and fluorescence was measured using the Canto II flow cytometer (BD Biosciences). Data were analyzed using FlowJo software (BD Biosciences).

### 4.5 MrkA structure determination by solution NMR spectroscopy

NMR spectra of ^13^C‐^15^N‐MrkA buffered in 50 mM sodium phosphate, 100 mM NaCl at pH 7.0 were acquired at 298 K on Avance 950 MHz Bruker spectrometers, processed using the standard Bruker software TOPSPIN, and analyzed through computer‐aided resonance assignment (CARA). The protocol for backbone and sidechain resonance assignment was described previously (Monaci et al., 2024). The assignment of the ^15^N‐edited nuclear overhauser effect (NOE) spectroscopy (NOESY) and ^13^C edited NOESY spectra allowed obtaining the NOEs, which were converted into distance restraints. Backbone dihedral angle constraints were derived from ^15^N, ^13^C’, ^13^C_α_, ^13^C_β_, and H_α_ chemical shifts, using the TALOS‐N program (Shen & Bax, 2013). Meaningful proton–proton distance restraints with *ϕ* and *ψ* backbone dihedral angle restraints were used for structure calculations. Distance restraints were considered meaningless and were not used in the calculations when they would be satisfied in all possible conformations or were between two hydrogen atoms at fixed distances (i.e., constraints between geminal hydrogen atoms). The CYANA tool (Güntert, 2004) was exploited to calculate MrkA family conformers and AMBER software (Case et al., 2005) to refine the final structure in explicit water solvent.

## AUTHOR CONTRIBUTIONS

**Valentina Monaci:** Investigation; methodology; writing – original draft. **Davide Oldrini:** Writing – review and editing. **Gianmarco Gasperini:** Writing – review and editing; conceptualization; supervision. **Lucia Banci:** Writing – review and editing. **Francesca Cantini:** Writing – review and editing; supervision; conceptualization; methodology. **Francesca Micoli:** Writing – review and editing; supervision; conceptualization.

## CONFLICT OF INTEREST STATEMENT

D.O., G.G., and F.M. are employees of the GSK group of companies. All other authors declare no competing interests. This work was sponsored by GlaxoSmithKline Biologicals SA. GSK Vaccines Institute for Global Health srl is an affiliate of GlaxoSmithKline Biologicals SA.

## Supporting information


**Table S1.** BLAST results of Pasteur Institute *Klebsiella* database.
**Table S2.** Protein sequences.
**Table S3.** Statistical analysis of the energy‐minimized family of MrkA conformers. KP

**Figure S1.** Number of meaningful NOEs per residue of MrkA. White, light, dark gray and black bars indicate intra‐residue, sequential, medium‐range and long range connectivities, respectively. The secondary structure elements are reported at the top.
**Figure S2.** RMSD values per residue (16‐175; 181‐201) to the mean structure for the backbone (filled squares) and all heavy atoms (filled circles) of the family of 20 conformers of MrkA after energy minimization. The secondary structure elements are also reported above.

## Data Availability

Coordinates have been deposited in the Protein Data Bank with accession number 9HW9; the resonance assignments were previously deposited in the Biological Magnetic Resonance Data Bank with accession code 52205.
