# Supplementary material for: Biological and structural characterization of the Type 3 fimbrial subunit MrkA from Klebsiella pneumoniae
Source: Protein Sci. 2025 Oct 21;34(11):e70343. doi: 10.1002/pro.70343 (PMC12539426; doi:10.1002/pro.70343)
Supplement: Supplementary file 2 — Figure S1. Number of meaningful NOEs per residue of MrkA. White, light, dark gray and black bars indicate intra‐residue, sequential, medium‐range and long range connectivities, respectively. The secondary structure elements are reported at the top. Figure S2. RMSD values per residue (16‐175; 181‐201) to the mean structure for the backbone (filled squares) and all heavy atoms (filled circles) of the family of 20 conformers of MrkA after energy minimization. The secondary structure elements are also reported above. [file PRO-34-e70343-s001.docx]

**Supplementary data**

**Table S2. Primers and protein sequences.**

| **Protein name** | **Coding sequence** |
| --- | --- |
| Self-complemented MrkA monomer | MGSHHHHHHHHHHGSCTVSVNGQGSDANVYLSPVTLTEVKAAAADTYLKPKSFTIDVSNCQAADGTKQDDVSKLGVNWTGGNLLAGATSKQQGYLANTEASGAQNIQLVLSTDNATALTNKIIPGDSTQPKAKGDASAVADGARFTYYVGYATSAPTTVTTGVVNSYATYEITYQGGGGGGADTTVGGGQVNFFGKVTDVS |
| KP3 mAb | VH:  QVQLQESGPGLVKPSETLSLTCTVSGGSMNSNSNTYYWGWIRQPPGKGLEWIGTIHSSGRTYYNPSLKSRVTISVDMSKNQFSLNLTSATAADTAVYYCARDLSGASLAPRRPFNYYYYNMDVWGRGTLVTVSS  VL:  DVVMTQSPLSLPVTLGQPASISCRSSQSLVYSDGNTYLNWFQQRPGQSPRRLIYKVSNRDSGVPDRFSGSGSGTDFTLKISRVEAEDVGVYYCMQGTHWPPITFGQGTRLEIK |
| St1C1 mAb | VH:  QVQLVQSGAEVRKPGASVTVFCRTSGYIFTSYAVHWVRQAPGQGLEWMGGINGGNGNTRISQRFQDRLMITRDRSANTASMELRSLTSEDTAIYYCARADDCSGVGCHPWFDPWGRGTLVTVSS  VL:  QSVLTQPPSVSVSPGHTASITCSGDKLGDKYVSWYQQKSGQSPVLVMYKDTKRPSGIPERFSGSNSGNTATLAISGTQAVDEADYFCQAWDRSIMIFGGGTKVTVL |
| St4C6 mAb | VH:  QVQLVQSGAEVRKPGASVTLSCRTSGYTFTSYAVHWVRQAPGQGLEWMGGVNGGNGNTRFSQKFQDRLMIVRDRSANTASMELRSLTSEDTAVYYCARADDCSGVGCHPWFDPWGQGTLVTVSS  VL:  SVILTQPPSVSVSPGQTANITCSGDKLGDKYTSWYLQKPGQSPVLLIFQDTKRPSDIPERFSGSNSGNTATLTISGTQAVDEADYYCQAWDSDSGTATFGGGTKLTVL |

**Table S3.** **Statistical analysis of the AMBER refined family of MrkA conformers.**

|  | **MrkA**  **(20 Conformers) ^a^** |
| --- | --- |
| Total number of meaningful NOE upper distance constraints | 2248 |
| **Intra-protein NOEs ^b^** | |
| Intra-residue | 434 |
| Inter-residue |  |
| Sequential (\|i-j\| = 1) | 683 |
| Medium-range (\|i-j\| > 1 and \|i-j\| < 5) | 257 |
| Long-range (\|i-j\| > 5) | 874 |
| Total meaningful dihedral angle restraints ^b^ | 269 |
| Phi | 134 |
| Psi | 135 |
| Number of restraints per residue | 12.5 |
| Number of long-range restraints per residue | 4.3 |
| **RMS violations per meaningful distance constraint (Å):** |  |
| Intraresidue | 0.0125±0.0020 |
| Sequential | 0.0125±0.0015 |
| Medium range | 0.0134±0.0013 |
| Long range | 0.0135±0.0013 |
| **RMS violations per meaningful dihedral angle constraints (°):** |  |
| Phi | 4.6872±2.0750 |
| Psi | 2.5860±1.1284 |
| **Violations** |  |
| > 0.5 Å | 0 |
| 0.1 - 0.2 Å | 4.0 |
| 0.2 - 0.5 Å | 0.1 |
| **Average RMSD to the mean (Å)** |  |
| Residue range 16-175;181-201 (backbone atoms) | 1.0800±0.2100 |
| Residue range 16-175;181-201 (all heavy atoms) | 1.4600±0.2100 |
| residual CYANA Target Function (Å^2^) | 1.20±0.13 |
| **Structural analysis** ^c^ |  |
| % of residues in most favorable regions | 72.20% |
| % of residues in allowed regions | 23.70% |
| % of residues in generously allowed regions | 0.90% |
| % of residues in disallowed regions | 0.20% |
| ^a^ Structure calculations were performed with the program CYANA 3.0 [1]. A total of 200 random conformers were subjected to 12000 steps of a simulated annealing process. All structure ensembles presented here were reﬁned with molecular dynamics using AMBER-16 [2]. Value of 50 kcal mol-1 Å^-2^ was used as force constants for the NOE and torsion angle restraints. The data are calculated over the 20 conformers representing the NMR structure.  ^b^ Number of meaningful constraints for each class. Backbone dihedral angle constraints were derived from ^15^N,^13^C’,^13^Cα,^13^Cβ, and Ha chemical shifts, using TALOS and added as restrains in the structure calculations as well.  ^c^ analyzed 80%. As it results from the Ramachandran plot analysis performed with PSVS [3]. | |

**Supplementary Figures**

**Figure S1. Number of meaningful NOEs per residue of MrkA.** White, light gray, dark gray and black bars indicate intra-residue, sequential, medium-range, and long-range connectivities, respectively. The secondary structure elements are reported at the top.
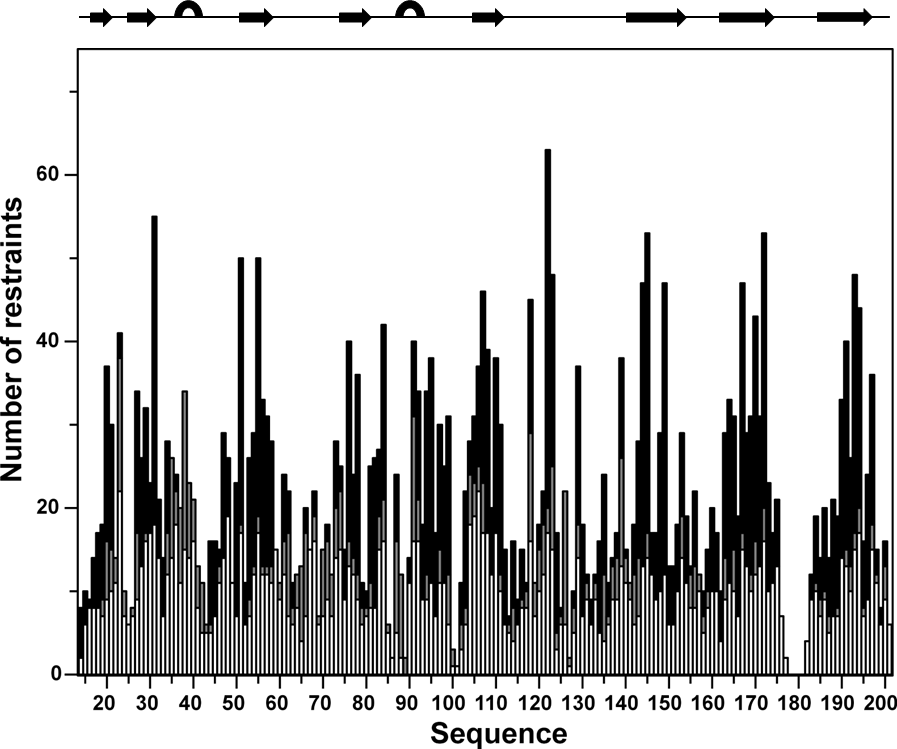


**Figure S2. RMSD values per residue (16-175; 181-201) to the mean structure for the backbone (filled squares) and all heavy atoms (filled circles) of the family of 20 conformers of MrkA after energy minimization. The secondary structure elements are also reported above.**

**
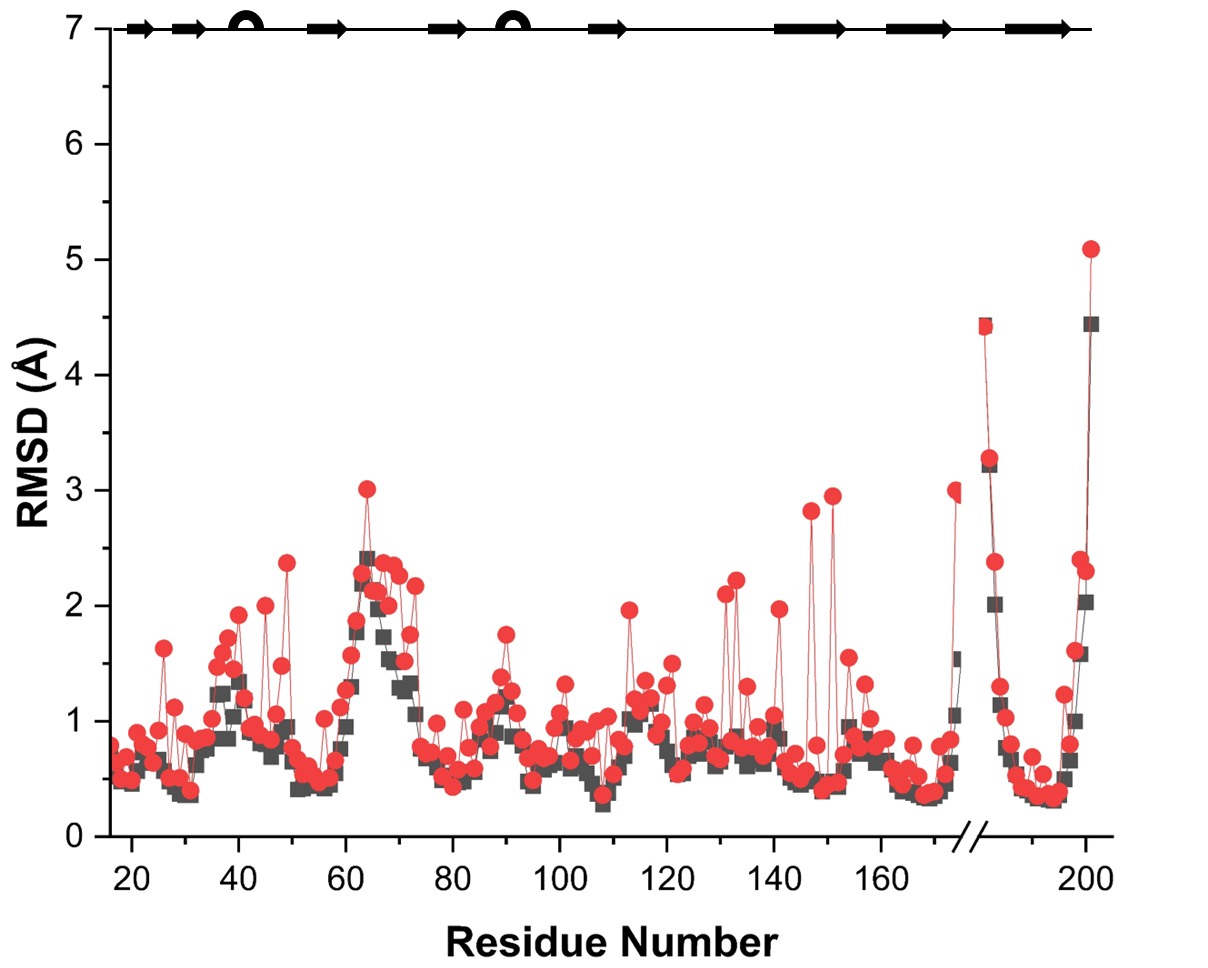
**
